# Effectiveness of an Educational Nursing Intervention on Caring Ability and Burden in Family Caregivers of Patients with Chronic Non-Communicable Diseases. A Preventive Randomized Controlled Clinical Trial

**DOI:** 10.17533/udea.iee.v37n1e04

**Published:** 2019-01-06

**Authors:** Myriam Duran Parra, Claudia Consuelo Torres, Ligia Betty Arboleda, Raquel Rivera Carvajal, Sherly Franco, Jenny Santos

**Affiliations:** 1 Nurse, M.Sc. Universidad de Santander, Bucaramanga, Colombia. email: enfermeria@udes.edu.co Universidad de Santander Universidad de Santander Colombia enfermeria@udes.edu.co; 2 Nurse, M.Sc. Universidad de Santander, Bucaramanga, Colombia. email: clau.torres@udes.edu.co Universidad de Santander Universidad de Santander Colombia clau.torres@udes.edu.co; 3 Nurse, M.Sc (c). Universidad de Santander, Bucaramanga, Colombia. email: libearsa@hotmail.com Universidad de Santander Universidad de Santander Colombia libearsa@hotmail.com; 4 MSc Nursing. Universidad de Santander, Bucaramanga, Colombia. email: raquelrivera_c@hotmail.com Universidad de Santander Universidad de Santander Colombia raquelrivera_c@hotmail.com; 5 B.Sc. Nursing student. Universidad de Santander, Bucaramanga, Colombia. email: sherlly_12@hotmail.com Universidad de Santander Universidad de Santander Colombia sherlly_12@hotmail.com; 6 B.Sc. Nursing student. Universidad de Santander, Bucaramanga, Colombia. email: Jnina_404@hotmail.com Universidad de Santander Universidad de Santander Colombia Jnina_404@hotmail.com

**Keywords:** noncommunicable diseases, chronic disease, caregivers, control groups, clinical trial., enfermedades no transmisibles, enfermedad crónica, cuidadores, grupos control, ensayo clínico., doenças não transmissíveis, doença crónica, cuidadores, grupos controle, ensaio clínico.

## Abstract

**Objective.:**

To evaluate the effect of the “Caring for Caregivers” program in the caring ability and burden in family caregivers of patients with chronic diseases at health care institutions.

**Methods.:**

A randomized controlled clinical trial was conducted in 34 relatives of patients with chronic diseases that had cared for them for more than 3 months. Zarit scale was used to measure caregiver burden and the CAI (Caring Ability Inventory) was also used to measure caring ability. An educational intervention was applied based on the “Caring for Caregivers” strategy of the Universidad Nacional de Colombia.

**Results.:**

Although both groups improved their percentage of unburdened caregivers from the first to the second assessment, the difference between the two assessments was 41.2% in the intervention group whereas it was 11.8% in the control group, being only statistically significant the difference for the intervention group. Regarding the caring ability, no significant changes were identified in both groups.

**Conclusion.:**

On family caregivers, it was observed that the “Caring for Caregivers” intervention had a positive impact on decreasing burden, but not on improving the caring ability.

## Resumen

**Objetivo.:**

Evaluar el efecto del programa “Cuidando a Cuidadores” en la habilidad del cuidado y la carga de los cuidadores familiares de personas con enfermedad crónica que asisten a una institución de salud.

**Métodos.:**

Ensayo clínico controlado randomizado, realizado en 34 familiares de personas con enfermedad crónica que los hubieran cuidado por más de 3 meses. Se utilizaron las escalas de ZARIT para medir la sobrecarga del cuidador y el CAI (*Caring Ability Inventory*) para medir la habilidad del cuidado. Se aplicó una intervención educativa basada en la estrategia “cuidando a cuidadores” de la Universidad Nacional de Colombia.

**Resultados.:**

Aunque ambos grupos mejoraron en el porcentaje de cuidadores sin sobrecarga de la primera a la segunda evaluación, en el grupo de intervención la diferencia entre los dos momentos de evaluación fue de 41.2%, mientras que en el grupo control fue de 11.8%, estadísticamente significante la diferencia para el grupo de intervención. En la habilidad del cuidado no se identificaron cambios significativos en los dos grupos.

**Conclusión.:**

En los cuidadores familiares se apreció que la intervención “cuidando a los cuidadores” presentó impacto positivo en disminución de la sobrecarga, pero no en la mejora de la habilidad de cuidado.

## Resumo

**Objetivo.:**

Avaliar o efeito do programa “Cuidando a Cuidadores” na habilidade do cuidado e a carga dos cuidadores familiares de pessoas com doenças crônica que frequentam a uma instituição de saúde.

**Métodos.:**

Ensaio controlado randomizado, realizado em 34 familiares de pessoas com doenças crônica que os tiveram cuidado por mais de 3 meses. Se utilizaram as escalas de ZARIT para medir a sobrecarga do cuidador e o CAI (Caring Ability Inventory) para medir a habilidade do cuidado. Se aplicou uma intervenção educativa baseada na estratégia “cuidando a cuidadores” da Universidade Nacional de Colômbia.

**Resultados.:**

Embora ambos grupos melhoraram na porcentagem de cuidadores sem sobrecarga da primeira à segunda avaliação, no grupo de intervenção a diferença entre os dois momentos de avaliação é de 41.2%, enquanto que no grupo controle é de 11.8%, sendo unicamente estatisticamente significante esta diferença para o grupo de intervenção. Na habilidade do cuidado não se identificaram câmbios significativos nos dos grupos.

**Conclusão.:**

Nos cuidadores familiares se apreciou que a intervenção “cuidando aos cuidadores” apresentou impacto positivo em diminuição da sobrecarga, mas não na melhora da habilidade de cuidado.

## Introduction

Chronic non-communicable diseases have an important impact on public health in Latin America and Colombia, being a big challenge for health personnel, especially for nursing, who should be involved to propose care interventions that meet with the General Health and Social Security System in a holistic and primary way.(1,2) In addition, chronic diseases are the leading causes of mortality and morbidity, which further indicate the need for care actions that aim at improving health care quality of patients and their families, who get affected by having to assume the burden generated by limitations, disabilities and dependence caused by chronic diseases, affecting the quality of life, especially of those caring for them.(3,4)Thus, chronic diseases cause disability and involve patients and their caregivers, that in most cases is a family member who assumes this function and does not receive any remuneration nor previous training, social support or any other service that prepare him/her to assume this new role. Most of them were have a low education level. These family caregivers require to be available without any time limitations, increase in costs, resources and efforts that may generate feelings of loneliness and emotional affectation due to the exhaustion that comes up from caring, generating a physical and mental impact on those who assume the caring role. All this leads to caregiver burden as a consequence of the combination of physical work, emotional pressure and economic burden.(5,6)

Regarding this aspect, the caring ability of patient caregivers plays a vital role and its impact on the survival of these patients and their disease management. Regarding this topic, the concept of ability is based on the holistic care of Milton Mayeroff,(7) described as the way to establish a relationship with another person that gets favored in his/her development. This author proposes a conceptual framework to study and understand the nursing care. In most cases, this implies understanding the person that receives care, considering their needs, strengths, weaknesses and whatever is involved in their well-being. The above also involves the knowledge of oneself, including beliefs and values, since these will be the base for the decisions that are made in relation to the patient. Thus, the caring ability is understood as the development of skills and abilities that are available to an individual to perform actions that help others to grow or appreciate their own life. 

Family caregivers should know the basics and be courageous and patient enough to deal with this situation,(6) but it requires to train and strengthening this ability to provide care that goes beyond care quality being offered to patients with chronic non-communicable diseases. This could also be an aspect that influences the well-being of people in charge of those chronicity situations.(8) In relation to burden, according to existing research,(9-12) nursing interventions to decrease burden have had a positive effect. However, these do not follow any methodology with a randomized control clinical trial that can clearly recommend the effect of these strategies in reducing the burden perception. Stress, fatigue, reduction in social relationship and all emotional disturbances affecting family caregivers converge on a burden in activities that impact the quality of life of caregivers, leading them to experience of coping with difficult situations at work, financial difficulties and to the point of affecting the family functioning, due to the fact that not only the main caregiver feels burdened but also those caregivers that occasionally get involved in activities.(7) 

The studies that include Colombia have implemented strategies to decrease this burden in relatives of patients with chronic non-communicable diseases such as the use of information technology and communication through counseling that seek an active listening and knowledge of the disease, which help decrease the levels of anxiety and depression that might be present in caregivers, resulting in a decrease in burden. This also contributes to improve access to health system information and have more contact with other caregivers dealing with a similar situation, which contributes to having a better perception of the disease and quality of life.(9)

Considering the important background of the studies conducted by the Chronic Care group of the Universidad Nacional and the researcher network in the field of caring for caregivers of patients with chronic diseases that have conducted multiple research on this topic, the “Caring for Caregivers” program has been developed with the aim of improving the caring ability and decreasing burden in family caregivers of patients with chronic non-communicable diseases. The caring group of Universidad de Santander (UDES) in partnership with the Latin American Network of Chronic Patients and their Families generated a proposal that helps guide the management of disease situations in both patients and their caregivers. For that, its main objective is to evaluate the effect of the “Caring for Caregivers” program in relation to the caring ability in family caregivers of patients with chronic diseases at a private health care institution in Bucaramanga. 

## Methods

A preventive controlled clinical trial was conducted with two comparison groups (intervention and control) in 2017. The inclusion criteria were to be aged 18 or over, be the caregiver of a patient with a chronic non-communicable disease (NCD) and the time devoted to caring was at least three months. Caregivers with difficulties in communication and those who could not receive the whole intervention were excluded. To meet the selection criteria, 34 family caregivers of patients with NCD were selected, who were users of a private tertiary hospital in Bucaramanga (Colombia).

### Randomization.

To assign the intervention, on behalf of the project coordination center, a co-researcher of this study randomized family caregivers in balanced blocks to either the control group or intervention group, by using a list of random numbers generated in Excel.

### Intervention.

The intervention consisted of the application of a four-topic workshop aimed at improving the family caregiver ability on knowledge, courage and patience, using a booklet designed by the Universidad Nacional de Colombia (13). This workshop was individually applied to each patient in a window of 3 hours at the hospital and was led by a nurse of the research team accompanied by a final year student of the nursing degree. The topics, objectives and activities are described in Table 1. Training related to the chronic pathology was provided to the control group, using educational support material designed by the research team. All participants of the control group were offered to have an intervention after the post-intervention surveys had been applied. 

**Table 1:** “Caring for Caregivers” Program Modules(13)

TOPICS	OBJTIVE	ACTIVITIES
INTRODUCTION	To introduce the topic and its recognition	Group members introduce themselves, including caregivers who start the program as well as those who coordinate it
KNOWLEDGE REQUIRED TO UNDERSTAND AND FACILITATE THE CARE- GIVER ROLE.	To generate a space for knowl- edge and recognition of the people involved in the family caring process around the expe- rience of chronic diseases	• Appreciation of the most beautiful and important aspects of the people involved in caring and the way they are expressed. • Aspects that take hard work and that inspire confidence to the caregiver. • Identification of caring activities done with oneself and others. • Solving questions related to the level of caring preparation.
SOCIAL ABILITY AND DECISION MAKING WHEN CAREGIVING	To apply a decision model and recognition of caregivers’ sup- port when caregiving	• Similarities among caregivers. • Coping with caring difficulties. • Identifying one’s own value. • Decision-making process applied in caregiving situations. • Strengthening of the social support network. Provision of sup- port and action in emergencies
EXPERIENCE OF GROW- ING AND UNDERSTAND- ING THE MEANING OF CARING	To reevaluate the experience of being a caregiver. To under- stand the meaning of patience as a growth element in the caring process	• Whatever calms down and exasperates the caregiver. • Identifying what caregivers can do to be more patient. • Setting goals to improve knowledge, courage and patience. • Skilled caregivers. • Caregivers with new goals and strategies. Recognizing oneself as a competent caregiver requiring help, guidance and rest. • Whatever will happen in the future and how to be prepared

### Instruments.

Zarit scale (Zarit Burden Interview)(14) was used to determine the perception of caregiver burden. This scale consists of 22 items with 5 Likert response options ranging from 1=never to 5=nearly always. The total score varies from 22 to 110, categorized as follows: no burden (≤ 46), mild burden (47-55) and severe burden (>55).(15) Its inter-observer reliability is 0.71 to 0.85 and an internal consistency using Cronbach’s alpha between 0.85 and 0.93.(11) This scale is widely used to measure caregiver burden.(16) To evaluate caring abilities, the Nkongho Caring Ability Inventory (17) was used in its Spanish version. The instrument consists of 37 questions answered on a Likert scale with scores from 1 to 7. Higher scores indicate a higher level of caring ability. Three dimensions are considered: knowledge (14 items), courage (13 items) and patience (10 items). This scale reports an internal consistency using Cronbach’s alpha between 0.84 and 0.86.(6,10) For the characterization of the family caregivers and patients, the GCPC-UN-D instrument for characterization was used for each caregiver-patient with chronic disease duo, in which demographic information was registered (age, marital status, sex, education level, origin, occupation and religion), as well as caregiver characteristics (time as caregiver, number of daily hours of care, and support available to the patient and caregiver.) In addition, this instrument contains the mental state assessment and the functional capacity assessment using SPMSQ and PULSES scales. The first instrument is a 10-questions questionnaire in which depending on the number of errors, it is categorized as intact mental functioning (0-2), mild cognitive impairment (3-4), moderate cognitive impairment (5-7) and severe cognitive impairment (8-10).(18) The PULSES (19) instrument evaluates the functional capacity of the patient to perform daily activities, in which there are 6 items scoring from 1 to 4, in which the higher score is obtained, the higher dependency. 

### Procedure and Information Collection.

The research team was responsible for the information collection (four nursing professionals, two of them hold a master’s degree in epidemiology and another one holds a postgraduate diploma in university teaching), with the collaboration of three students of the CUIDEN research seedbed group. All these people had prior training on the use of the instrument and the application of the intervention using the educational booklet “Caring for Caregivers”. Initially, hospitalized patients with chronic non-communicable diseases were identified at “Los Comuneros” University Hospital in Bucaramanga. Once the patient had been selected, the caregiver was identified to later define the objective, importance and procedures of the study. Then, the informed consent was signed and the instruments for the collection of sociodemographic information and the caring ability and burden assessment (pre-intervention) were filled out. Later, an educational workshop was carried out on the four topics of the “Caring for Caregivers” program to the intervention group. Likewise, an educational meeting on chronic diseases and their caring was carried out to the control group. Family caregivers were contacted on the next day to apply again the instruments of caring ability and burden assessment (post-intervention). 

People who carried out the study assessments and the responsible person for the statistical analysis of the information did not know to which group the participant’s information belonged. It was not possible to mask the group allocation due to the nature of the intervention. The process carried out with the group participants is shown in Table 2. 

**Table 2:** Tasks carried out in the study groups

GROUPS		DESCRIPTION
INTERVENTION	Control	
ENROLLMENT	Enrollment	Caregivers that met with the selection criteria were identified in
MEASUREMENT OF CARING ABILITY AND BURDEN	Measurement of caring abil- ity and burden	All caregivers were first measured for caring ability and burden, also the completion of the caregivers and patients’ characterization form.
AN EDUCATIONAL WORKSHOP WITH FOUR TOPICS CONTAINED IN THE “CARING FOR CAREGIVERS” BOOKLET.	An educational meeting with contents of pathology man- agement of the patient, the educational booklet designed by the research team.	Workshops were carried out by research team members in a single day, based on the prior group allocation and using either the “Caring for Caregivers” educational booklet that lasted around 3 hours or the educational booklet with basic chronic pathology management for patients that lasted around 30 minutes.
FINAL RESEARCH MEETING	Final research meeting	Later, instruments were applied again to measure the caring ability and burden of family caregivers after the intervention.

### Information Analysis.

The collected information was analyzed using the statistical software Stata14 (Stata Corporation, College Station, USA). Quantitative variables showed a nonparametric distribution, so the middle value and the interquartile range were reported. The Mann-Whitney U test was applied to identify differences among the groups (intervention and control groups). Relative and absolute frequencies were calculated for the categorical variables. Fisher’s exact test was also applied as cells had an expected value of less than 5. For all tests, when the reported probability value was less than 0.05, a statistical significance was assumed. To measure changes in scores of Zarit and CAI scales prior and after the intervention, the Wilcoxon signed-rank test was used for repeated measures in groups and the Mann-Whitney U test was used when assessing differences between groups.

### Ethical Principles.

This research received the approval from the bioethics committee of the Universidad de Santander and the Health Institution committee where the research was conducted. Participants signed an informed consent form. This project was registered in the Clinicaltrials.gov site under the number NCT03159728, the database for the record and the results of the clinical worldwide studies involving human subjects as participants. 

## Results

**Figure 1:**
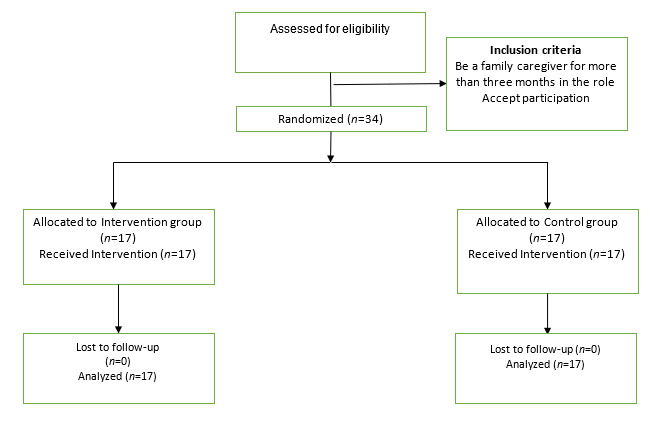
Flow diagram of the process of a randomized clinical trial in two parallel groups

The total number of participants was 34 family caregivers (17 in the intervention group and 17 in the control group). All people who agreed to participate were analyzed in the group that they were initially allocated. (Figure 1)

Sociodemographic characteristics are described in Table 3. It was observed that there are no significant differences in the demographic and support variables that might interfere with their comparability, except for the caregivers’ socioeconomic status, in which 58.82% of the caregivers in the intervention group are from the lowest strata while it was 11.76% in the control group. 

In general, patients with chronic non-communicable diseases, regardless of their group, can be described as mainly married individuals over 70 years of age with no or little education from low socioeconomic status. Caregivers are described as mostly women almost 30 years younger than patients, with secondary and higher education in a civil partnership and also from the lowest strata. As for the support that patients and their caregivers receive, no statistically significant difference was found. In addition, it can be observed that psychological and social support are the lowest supports in patients and caregivers in both groups, while religious, economic, and family support were the highest supports (the latter in all participants.)

**Table 3:** Characteristics of patients and family caregivers in each group

*Characteristics*		*Patients*			*Caregivers*	
	**Intervention *n*=17**	**Control *n*=17**	*p*	**Intervention *n*=17**	**Control *n*=17**	*p*
*Age; middle value (IQR)*	73 (69-88)	77 (72-80)	0.69	47 (40-58)	54 (48-56)	0.09
*Female gender; n (%)*	8 (47.06)	11 (64.71)	0.49	15 (88.24)	17 (100)	0.48
*Level of education; n (%)*			0.53			0.42
*None*	1 (5.88)	0		0	1 (5.88)	
*Primary education*	14 (82.35)	13 (76.47)		4 (23.53)	5 (29.41)	
*Secondary education*	2 (11.76)	2 (11.76)		10 (58.82)	5 (29.41)	
*Undergraduate degree*	0	2 (11.76)		1 (5.88)	3 (17.65)	
*Technical degree*	0	0		2 (11.76)	2 (11.76)	
*Postgraduate degree*	0	0		0	1 (5.88)	
*Marital status; n (%)*			0.69			0.10
*Single*	4 (23.53)	3 (17.65)		9 (52.94)	4 (23.53)	
*Married*	5 (29.41)	9 (52.94)		4 (23.53)	9 (52.94)	
*Separated*	2 (11.76)	1 (5.88)		0	2 (11.76)	
*Widowed*	5 (29.41)	4 (23.53)		4 (23.53)	2 (11.76)	
*Civil partnership*	1 (5.88)	0		0	0	
*Socioeconomic status; n (%)*			0.10			0.02
*1*	10 (58.82)	3 (17.65)		10 (58.82)	2 (11.76)	
*2*	4 (23.53)	5 (29.41)		4 (23.53)	5 (29.41)	
*3*	1 (5.88)	4 (23.53)		1 (5.88)	6 (35.29)	
*4*	2 (11.76)	4 (23.53)		2 (11.76)	4 (23.53)	
*6*	0	1 (5.88)		0	0	
*Socioeconomic status; n (%)*			0.10			0.02
*Residence sector; n (%)*			0.60			1
*Rural*	1 (5.88)	3 (17.65)		1 (5.88)	1 (5.88)	
*Urban*	16 (94.12)	14 (82.35)		16 (94.12)	16 (94.12)	
*Occupation; n (%)*			0.28			0.46
*Homemaker*	8 (47.06)	4 (23.53)		10 (58.82)	14 (82.35)	
*Employee*	0	1 (5.88)		1 (5.88)	2	
*Self-employed*	0	0		3 (17.65)	1 (5.88)	
*Other*	9 (52.94)	12 (70.59)		3 (17.65)	2 (11.76)	
*Support; n (%)*						
*Psychological*	2 (11.76)	7 (41.18)	0.06	1 (5.88)	5 (29.41)	0.07
*Family*	17 (100)	17 (100)	1	17 (100)	17 (100)	1
*Economic*	14 (82.35)	16 (94.12)	0.30	14 (82.35)	14 (82.35)	1
*Religious*	12 (70.59)	14 (82.35)	0.34	10 (58.82)	15 (88.24)	0.052
*Social*	7 (41.18)	7 (41.18)	1	12 (70.59)	9 (52.94)	0.481

Characteristics of patient caring in each group are shown in Table 4. No statistically significant difference was found among both groups. Regardless of the group, it was predominant the devotion of 24 hours of care per day required by the patient, the relationship with the patient is parent-son in seven out of ten cases, being the sole caregiver in the same proportion. The patient’s dependency level in the PULSES assessment is high and the mental impairment assessment using the SPMSQ scale is from moderate to severe in 23.5% of patients in the study group versus 41.2% in the control group.

**Table 4:** Characteristics of Patient Caring by Group

Characteristics		Group	
	**Intervention *n*=17**	Control n=17	*p*
Care hours per day Middle value (IQR)	*n*=17 24 (12-24)	*n*=17 24 (12-24)	0.50
**Relationship to the patient; *n* (%)**			1
Husband/Wife	2 (11.76)	3 (17.65)	
Son/Daughter	12 (70.59)	11 (64.71)	
Friend	1 (5.88)	1 (5.88)	
Other	2 (11.76)	2 (11.76)	
**Sole caregiver; *n* (%)**	12 (70.59)	12 (70.59)	1
**Patient PULSES Assessment; *n* (%)**			1
Low malfunction	1 (5.88)	1 (5.88)	
Partial dependent	4 (23.53)	3 (17.65)	
Total dependent	12 (70.59)	13 (76.47)	
**Patient SPMSQ Assessment; *n* (%)**			0.58
Intact	4 (23.53)	3 (17.65)	
Mild cognitive impairment	9 (52.94)	7 (41.18)	
Moderate cognitive impairment	3 (17.65)	3 (17.65)	
Severe cognitive impairment	1 (5.88)	4 (23.53)	

### Caregiver Burden

Although both groups improved their percentage of unburdened caregivers from the first to the second evaluation, the difference among the two evaluations is 41.17% in the intervention group while it was 11.76% in the control group, being only statistically significant the difference for the intervention group. When analyzing the data continuously, middle values in the control group were higher and varied than in the intervention group: The initial middle value was 66 in the control group (IQR: 50 - 77) and 53 in the intervention group (IQR: 49 - 62). When evaluating the change of these values, it is statistically significant in both groups, with probability values under 0.01. To evaluate differences among the groups, the final evaluation was subtracted from the initial one. The middle value for the lower half in the intervention group was 7 (IQR: -0.5 - 11) and 5.5 in the control group (IQR: 2.5 - 10), with a probability value of 0.9548, which indicated that the difference was not statistically significant. 

**Table 5:** Level of Caregiver Burden Based on the Study Group and Time of Evaluation

*Group*	*Evaluation*		*Burden Level*		
		Intense	Mild	Unburden	***p*-value**
		***n* (%)**	***n* (%)**	***n* (%)**	
*Intervention*	Initial	8 (47.06)	6 (35.29)	3 (17.65)	0.001
	Final	3 (17.65)	4 (23.53)	10 (58.82)	
*Control*	Initial	12 (67.71)	3 (17.65)	3 (17.65)	0.15
	Final	11 (64.71)	1 (5.88)	5 (29.41)	

### Caring Ability

It can be observed in Table 6 that the development of abilities did not show a statistically significant change in the levels of total ability and its dimensions, for both caregivers in the control group and intervention group. Within the evaluated dimensions, patience stands out to have the highest percentages of well-classified ones in the category of low ability in both groups, with percentages from 47.06% to 76.47%. 

**Table 6:** Caregivers Ability Based on the Study Group and Time of Evaluation

*Characteristic*	***Intervention (*n*=17)***		**p*-value***	***Control (*n*=17)***		*p-value*
	**Prior *n* (%)**	**Post *n* (%)**		**Prior *n* (%)**	**Post *n* (%)**	
*Full ability*			0.15			0.23
*Low ability*	3 (17.65)	3 (17.65)		2 (11.76)	4 (23.53)	
*Average ability*	5 (29.41)	4 (41.18)		5 (29.41)	5 (29.41)	
*High ability*	9 (52.94)	7 (41.18)		10 (58.82)	8 (47.06)	
*Knowledge*			0.44			0.91
*Low ability*	3 (17.65)	4 (23.53)		2 (11.76)	2 (11.76)	
*Average ability*	6 (35.29)	6 (35.29)		5 (29.41)	6 (35.29)	
*High ability*	8 (47.06)	7 (41.18)		10 (58.82)	9 (52.94)	
*Courage*			0.07			0.14
*Low ability*	1 (5.88)	3 (17.65)		2 (11.76)	1 (5.77)	
*Average ability*	3 (17.65)	7 (41.18)		2 (11.76)	9 (52.94)	
*High ability*	13 (76.47)	7 (41.18)		13 (76.47)	7 (41.18)	
*Patience*			0.25			0.65
*Low ability*	10 (58.82)	13 (76.47)		8 (47.06)	10 (58.82)	
*Average ability*	5 (29.41)	2 (11.76)		6 (35.29)	3 (17.65)	Average ability
*High ability*	2 (11.76)	2 (11.76)		3 (17.65)	4 (23.53)	High ability

When analyzing the variable continuously, the values for caring ability decreased in both groups (from 223 (IQR: 207 - 242) to 214 (IQR: 202 - 223) in the intervention group and from 236 (IQR: 214 - 242) to 212 (IQR: 205 -235) in the control group), being this difference at the borderline of the statistical significance in the intervention group (*p*=0.0467)**.** In relation to the CAI scale dimensions, only the scores of the courage dimension had a significant difference in the intervention group (from 83 (IQR: 75 - 85) in pre-intervention and 74 (IQR: 67 - 76) in post-intervention), which corresponds to a probability of 0.0128, showing that these might have influenced on the full ability scores to have these unfavorable changes. 

## Discussion

Caregivers are mostly women aged between 40 and 58 years old, similar to the findings of other studies.(20-24) The occupation with the highest percentage is being a homemaker, with 58.82% in the intervention group and 82.35% in the control group. This differs from the study published by Arias *et al*. in 2014, with individuals from different regions of Colombia in which the homemaker occupation was reported to be between 35.71% and 57%.(25)

Patients presented high dependency levels with 70.59% in the intervention group and 76.47% in the control group, which is related to the intense caregiver burden.(26) These levels differ somewhat from those found by Vega *et al*.(21) in which 48% of patients had a severe dependency, 30% with moderate dependency and 22% mild dependency. Moderate or severe mild cognitive impairment was 23.53% in the intervention group and 41.18 % in the control group, which is different from the study conducted by Soto *et al*. in which 52.9% were identified to present this feature but in this study, patients from hospital palliative care units in Spain were only involved. The level of cognitive impairment and patient dependency were determining factors for keeping elderly patients active,(27) having an impact on caregiver burden.

In previous quasi-experimental studies, important findings on the effect of intervention have been reported, such as the improvement on the specific ability of knowledge dimension, in which post-intervention changes have resulted to be very favorable. In addition, the intervention has been reported to have a positive effect on the knowledge and patience dimensions, but it did not have any on the courage dimension.(28,29) In our study, there were no favorable changes for the caring ability. On the contrary, courage dimension degraded in particular, which may mean that the strategy used may not be convenient. At one end, the training of these topics in a single session may be exhausting and at the other end, if the session is held individually, caregivers will not be able to share their experiences, as it happens in group workshops where they can realize what happens to other people that are in similar situation. A similar result was reported by Vega *et al*.(21) in which a decrease in the average of the courage dimension was observed, especially in the control group. In addition, the study published by Montalvo *et al*. (30) reported that courage was the dimension with the lowest score at the beginning and end of the program and that despite the changes in the caregiver ability, these were not statistically significant. It was relevant that the patience dimension had the highest percentages in the categories of low ability level for both intervention group and control group, and that it has also been reported Chaparro *et al.*(31)

With regards to burden, a study with elderly patients to evaluate the effect of an educational intervention showed an important burden reduction between pre-test and post-test, demonstrating that interventions have a positive effect on this variable.(32) This finding is very similar to that found in our study in which there was an increase in the percentage of unburdened caregivers from the first to the second evaluation (41% in the intervention group versus 12% in the control group, when the variable is categorically analyzed) When burden analysis is carried out continuously, the effect on the control group can be observed, which might be explained by the fact that these caregivers tend to live in places with higher socioeconomic levels (82.35% of the intervention group caregivers used to live in stratum 1 and 2 as well as 41.17% of the control group caregivers), and could access the different support types. Therefore, control group caregivers tend to report higher percentages of psychological and religious support, compared to the reported by the intervention group, in 29.41% and 88.24% for each dimension, while these were 5.88% and 58.82% respectively in the intervention group. In addition, it is important to consider that the intervention in the control group might have an impact, since information related to the patient pathology was provided, such as risk factors, possible complications, preventive measures for pathology management, among others.

Perceived burden levels are similar to the those reported in by Leal *et al*.(33) in which the implementation of an educational program in three groups of relatives of schizophrenic patients that had previously participated in different educational activities found out that the intervention had a beneficial effect in reducing the proportion of relatives with burden perception. Burden levels were different to those in the study published by Eterovic *et al*.(34) in which 13.9% reported an intense burden and its population was bedridden people of different ages with severe disability or loss of autonomy.

In conclusion, the educational intervention “Caring for Caregivers” proved to be effective in decreasing the burden perception on caregivers but it was not conclusive on the changes generated in the caring ability in caregivers of patients with chronic non-communicable diseases. A limitation of the study was the difficulty to meet the inclusion criteria for time caring for the patient. In addition, considering that caregivers have their relatives in hospital at the time, they did not have much time to attend the educational intervention. However, it was possible to intervene during their stay in the hospital. Considering the above two limitations and the results presented, it was decided to continue with the second part of this study by developing group workshops with aim of doing this intervention in 1 or 2 sessions, as the limitation for caregivers to attend the total sessions is still present.

## References

[1] Vega-Angarita O, Mendoza-Tarazona M, Ureña-Molina M, Villamil Santander W (2008). Efecto de un programa educativo en la habilidad de cuidado de los cuidadores familiares de personas en situación crónica de enfermedad. Rev. Cienc. Cuid.

[2] Barreto-Osorio RV, Campos de Aldana MS, Carrillo-González GM, Coral-Ibarra R, Chaparro-Díaz L, Durán-Parra M (2015). Entrevista Percepción de Carga del Cuidado de Zarit: pruebas psicométricas para Colombia. Aquichan.

[3] Organización Mundial de la Salud (2012). Estrategia para la prevención y el control de las enfermedades no transmisibles, 2012- 2025.

[4] Ávila NL, Flores MM, Santos MT, Ochoa MC, Gallegos A (2012). Sobrecarga en el cuidador de paciente con infarto agudo al miocardio. Rev. Cuid.

[5] Sánchez RT, Molina EM, Gómez-Ortega OR (2016). Intervenciones de enfermería para disminuir la sobrecarga en cuidadores: un estudio piloto. Rev. Cuid.

[6] Ortiz C, Grecia Yirle (2013). Características sociodemográficas asociadas a la sobrecarga de los cuidadores de pacientes diabéticos en Cúcuta. Rev. Cuid.

[7] Mayeroff M (1972). On Caring. J. Philos.

[8] Barrera OL, Galvis CR, Moreno ME, Pinto AN, Pinzón ML, Romero GE, Sanchez HB (2006). La habilidad de cuidado de los cuidadores familiares de personas con enfermedad crónica. Invest. Educ. Enferm.

[9] Carrillo G, Sánchez B, Barrera L (2015). Habilidad de cuidado de cuidadores familiares de niños con cáncer. Rev. Salud Pública.

[10] Hernández N, Moreno C, Barragán J (2014). Necesidades de cuidado de la diada cuidador-persona. Expectativa de cambio en intervenciones de enfermería. Rev. Cuid.

[11] Eterovic C, Mendoza S, Sáez K (2015). Habilidad de cuidado y nivel de sobrecarga en cuidadores informales de personas dependientes. Enferm. Global.

[12] Sánchez B, Chaparro L, Carrillo GM (2016). La carga del cuidado en la enfermedad crónica en la díada cuidador familiar-receptor del cuidado. Invest. Educ. Enferm.

[13] Barrera L, Pinto N, Sánchez B, Carrillo M, Chaparro L (2013). Cuidando a los Cuidadores familiares de personas con enfermedad crónica Cartilla. Bogotá: Grupo de cuidado al paciente crónico y la familia.

[14] Zarit SH, Reever KE Back-Peterson J (1980). Relatives of the impaired elderly: correlates of feelings of burden. Gerontologist.

[15] Abreu W, Tolson D, Jackson GA, Costa N (2018). A cross-sectional study of family caregiver burden and psychological distress linked to frailty and functional dependency of a relative with advanced dementia. Dementia.

[16] Arroyo E, Arana A, Garrido R, Crespo R (2018). Análisis de la sobrecarga del cuidador del paciente en diálisis. Enferm. Nefrol.

[17] Nkongho N (1990). The Caring Ability Inventory. In: Strickland OL, Waltz CF. Measurement of Nursing Outcomes: Self Care and Coping.

[18] Martínez J, Dueñas R, Carmen Onís M, Aguado C, Albert C, Luque R (2001). Adaptación y validación al castellano del cuestionario de Pfeiffer (SPMSQ) para detectar la existencia de deterioro cognitivo en personas mayores de 65 años. Med. Clin (Barc).

[19] Marshall S, Heisel B, Grinnell D (1990). Validity of the PULSES Profile Compared Whit the Functional Independence Measure for Measuring Disability in a Stroke Rehabilitation Setting. Arch. Phys. Med. Rehabil.

[20] Hu XL, Dolansky MA, Hu XY, Zhang FY, Qu MY (2016). Factors associated with the caregiver burden among family caregivers of patients with heart failure in Southwest China. Nurs. Health Sci..

[21] Vega OM, González DS, Ramírez MM (2009). Fortalecimiento del cuidado a cuidadores de personas con enfermedad crónica en red: resultados del estudio multicéntrico en el Norte de Santander. Respuestas.

[22] Alonso A, Garrido G, Díaz A, Casquero R, Riera M (2004). Perfil y sobrecarga de los cuidadores de pacientes con demencia incluidos en el programa ALOIS. Aten. Primaria.

[23] Valle-Alonso De, Hernández-López I, Zúñiga-Vargas M, Martínez-Aguilera P (2015). Sobrecarga y Burnout en cuidadores informales del adulto mayor. Enferm. Univ.

[24] Campos de Aldana MS, Moya D, Mendoza JD, Duran EY (2014). Las enfermedades crónicas no transmisibles y el uso de tecnologías de información y comunicación: revisión sistemática. Rev. Cuid.

[25] Arias M, Barrera L, Carrillo GM, Chaparro L, Sánchez B, Vargas E (2015). Cuidadores familiares de personas con enfermedad crónica en las regiones de frontera colombiana: perfil y carga percibida de cuidado. Rev. Fac. Med.

[26] Soto-Rubio Ana, Pérez-Marín Marián Antonia, Barreto Pilar (2017). Frail elderly with and without cognitive impairment at the end of life: their emotional state and the wellbeing of their family caregivers. Arch. Gerontol. Geriatr.

[27] Salazar-Barajas ME, Lillo CM, Hernández CPL, Villarreal RMA, Gallegos CE, Gómez MM, Salazar- González BC (2018). Factors Contributing to Active Aging in Older Adults, from the Framework of Roy’s Adaptation Model. Invest. Educ. Enferm.

[28] Díaz-Álvarez Juan Carlos, Rojas-Martínez MV (2009). Cuidando al cuidador: efectos de un programa educativo. Aquichan.

[29] Morales-Padilla O (2008). Efectos del Programa Cuidando al Cuidador, en la mejora de la habilidad de cuidado. Av. Enferm.

[30] Montalvo-Prieto M, Flórez-Torres I, Stavro D (2008). Cuidando a cuidadores familiares de niños en situación de discapacidad. Aquichan.

[31] Chaparro L, Carreño SP, Campos-de-Aldana MS, Benavides F, Niño- Cardozo CL, Cardona RM (2016). La habilidad de cuidado del cuidador familiar en diferentes regiones de Colombia. Rev. UDCA Actual. Divulg. Cient.

[32] Velásquez V, López L, Cataño N, Muñoz E (2011). Efecto de un programa educativo para cuidadores de personas ancianas. Una perspectiva cultural. Rev. Salud Pública.

[33] Leal M, Sales R, Ibáñez E, Giner J, Leal C (2008). Valoración de la sobrecarga en cuidadores informales de pacientes con esquizofrenia antes y después de un programa psicoeducativo. Actas Esp. Psiquiatr.

[34] Eterovic C, Mendoza S, Sáez K (2015). Habilidad de cuidado y nivel de sobrecarga en cuidadoras/es informales de personas dependientes. Enferm. Glob.

